# Exploring Cryoglobulinemia's Clinical Odyssey: A Case Series

**DOI:** 10.1002/jha2.70029

**Published:** 2025-04-02

**Authors:** Shivangini Duggal, Lakshmi Vaishnavi Prasanna Kattamuri, Michel Toutoungy, Eder Luna Ceron, Madhumita Rondla, Angelica Lehker

**Affiliations:** ^1^ Department of Internal Medicine Texas Tech Health Sciences Center El Paso Texas USA; ^2^ Paul L. Foster School of Medicine Texas Tech Health Sciences Center El Paso Texas USA

**Keywords:** case series, colon cancer, cryoglobulinemia, hepatitis C, monoclonal gammopathy of undetermined significance

## Abstract

Cryoglobulinemia (CG) encompasses disorders in which immunoglobulins precipitate at low temperatures. According to Brouet's classification, Type I CG is linked to plasma cell disorders, while mixed CG (Types II and III) is associated with autoimmune conditions, infections (notably hepatitis C virus [HCV]), and lymphoproliferative disorders. Each type presents distinct symptoms, with Type I often causing severe vasomotor symptoms and Types II and III involving systemic features like purpura, Raynaud's phenomenon, and renal involvement. This case series presents three CG cases, illustrating diverse etiologies and presentations. Case 1 discusses CG in metastatic colon cancer with *Staphylococcus aureus* bacteremia, highlighting infection‐triggered CG. Case 2 covers HCV‐related mixed CG, emphasizing antiviral therapy's role. Case 3 describes a CG flare after rituximab therapy, managed with steroids. These cases emphasize a multidisciplinary approach and individualized management to address CG's complexity and improve patient outcomes.

**Clinical Trial Registration**: The authors have confirmed clinical trial registration is not needed for this submission.

## Graphical abstract

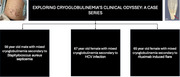

## 1 Introduction

Cryoglobulinemia (CG) is a pathological condition characterized by the precipitation of circulating immunoglobulins when serum is cooled below 4°C, a process that is reversible upon rewarming. First described by Wintrobe and Buell in 1933, cryoglobulins were later identified as gamma globulins by Lerner and Watson, who introduced the term cryoglobulins to describe these cold‐precipitable serum proteins [1, 2]. Since then, the understanding of CG has evolved significantly, particularly after the classification proposed by Brouet et al. in 1974, which categorized cryoglobulins into three types based on their immunochemical composition: type I, consisting of monoclonal immunoglobulins (typically IgM, IgG, or IgA); type II, formed by monoclonal IgM with rheumatoid factor activity in combination with polyclonal IgG; and type III, comprising polyclonal IgM and IgG [3]. Over time, it has become clear that CG essentially consists of two major forms. Type I is associated with hemostasis disorder accompanied by multiple thromboses of small and medium‐sized vessels driven by hematological malignancies. Mixed CG (types II and III) represents an inflammatory small‐vessel vasculitis caused by complement‐mediated immune‐complex deposition often linked to chronic infections (hepatitis C), systemic autoimmune diseases, or indolent B‐cell lymphoproliferation [4].

Cryoglobulinemic syndrome encompasses a broad spectrum of clinical manifestations, ranging from mild symptoms such as purpura and arthralgia to severe, life‐threatening organ involvement [5]. Common features across all forms include vascular purpura, arthralgia, peripheral neuropathy, and renal involvement. However, distinct differences exist between type I and mixed CG. Type I CG primarily presents with hyperviscosity‐related symptoms, including Raynaud's phenomenon, acrocyanosis, digital ischemia, and thrombotic events [6, 7, 8], whereas mixed CG is associated with systemic vasculitis affecting the skin, joints, kidneys, and peripheral nerves [9, 10]. Neuropathy in type I CG is due to ischemic neuropathy from vascular occlusion, whereas mixed CG leads to sensory‐motor polyneuropathy or mononeuritis multiplex [6, 7, 8, 9, 10].

The diagnosis of CG relies on the detection of cryoglobulins in serum, which requires meticulous handling of blood samples. Serum must be maintained at 37°C until separation to prevent premature cryoprecipitation. Further immunophenotyping determines the cryoglobulin type, guiding the etiologic assessment. Additional laboratory tests, including serum protein electrophoresis, immunofixation, rheumatoid factor, complement levels (particularly C4), and viral serologies, are essential for identifying underlying causes. In cases where cryoglobulins are not readily detected despite strong clinical suspicion, tissue biopsies (e.g., skin, kidney, or nerve) may confirm the diagnosis [11, 12, 13].

Here, we present a case series of three patients with distinct clinical etiologies leading to CG, highlighting the diverse underlying conditions, clinical presentations, and diagnostic challenges associated with this syndrome (Table 1).

**TABLE 1:** Summary of three cases of cryoglobulinemia, highlighting patient demographics, clinical presentation, underlying conditions, cryoglobulinemia cause and type, management strategies, and recommended follow‐up care.

Demographics	Presenting complaint	Past medical history	Cause of cryoglobulinemic syndrome	Type of cryoglobulinemia	Treatment provided	Follow‐up provided
56‐year‐old Hispanic male	Left‐sided chest pain, low‐grade fever, purpuric rash	Stage IV colon cancer with liver and lung metastases, on FOLFOX	*Staphylococcus aureus* septicemia	Mixed cryoglobulinemia (associated with infection)	Cefazolin for 6 weeks, port‐a‐cath removal	Hematology‐oncology, rheumatology and GI
67‐year‐old female	Severe fatigue, worsening arthralgias, palpable purpura	Hepatitis C genotype 1a‐related cirrhosis, hepatic steatosis, fibrosis (F4), HCV treatment discontinued	Chronic HCV infection and no response to initial treatment	Mixed cryoglobulinemia (HCV‐related)	Restarted on HCV treatment	Rheumatology and infectious disease
65‐year‐old female	Severe fatigue, palpable purpura (lower extremities)	MGUS, type II CG vasculitis (IgM+), MPGN, and recent rituximab use	Rituximab‐induced cryoglobulinemic flare	Mixed cryoglobulinemia (cryoglobulinemic flare)	Pulse‐dose steroids	Nephrology and hematology‐oncology

## 2 Case Reports

### 2.1 Case 1

**FIGURE 1:**
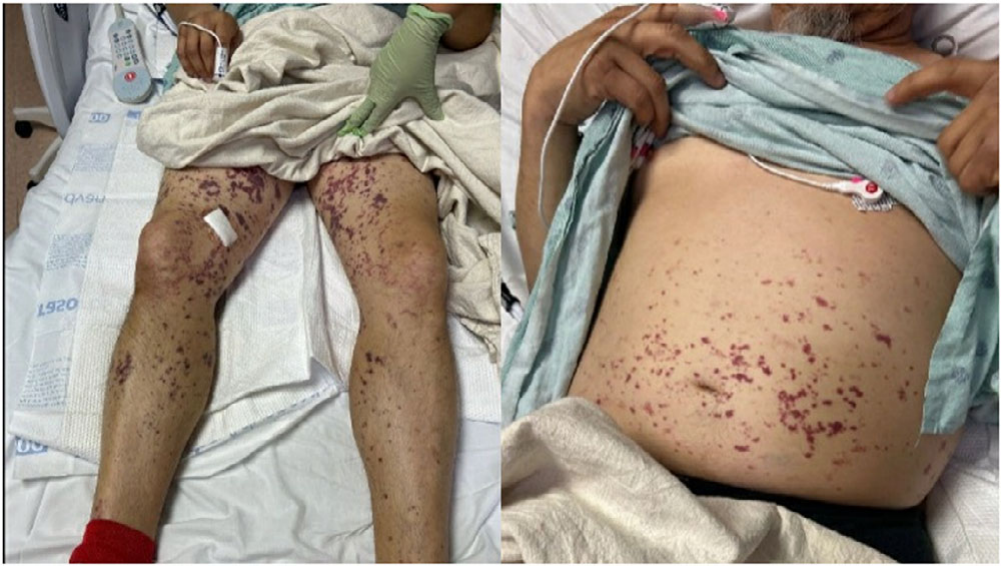
Displaying the palpable purpura on the extremities and abdomen for Case 1.

A 56‐year‐old Hispanic male with a past medical history of stage IV colon cancer metastatic to the liver and lungs presented with left‐sided chest pain and low‐grade fever. He was undergoing FOLFOX chemotherapy for colonic adenocarcinoma through a port‐a‐cath. Physical examination revealed inflammation around the port‐a‐cath site and diffuse non‐pruritic palpable purpuric rash mostly involving the trunk and lower extremities. Significant labs are illustrated in Table 2. Patient underwent removal of the port‐a‐cath and blood cultures revealed *Staphylococcus aureus* septic arthritis involving the left sternoclavicular joint along with a left subclavian vein thrombosis. Skin biopsy taken from the rash (Figure 1) on the extremities was suggestive of acute vasculitis. Further investigation revealed a low complement (C4 12.4 mg/dL) and positive cryocrit (2%), implying that he had CG. He received a six‐week course of cefazolin for treatment of septic arthritis and *S. aureus* septicemia and suspected infective endocarditis (IE). Treatment with cefazolin showed resolution of his CG as it was secondary to infectious causes. No adverse effects were reported. He was discharged outpatient with follow‐up for hematology‐oncology, rheumatology and gastroenterology.

**TABLE 2:** Laboratory Workup for Case 1.

Serum test [Normal Range]	Results on initial presentation	Results on discharge
WBC [(4.50–11.00) × 10^3^/UL]	17.17 × 10^3^/UL	3.92 × 10^3^/UL
RBC [(4.20–5.90) × 10^6^/UL]	4.91 × 10^6^/UL	2.51 × 10^6^/UL
HGB [12.0–16.0 g/dL]	16.1 g/dL	8.3 g/dL
HCT [38.0%–47.0%]	46.4%	24.8%
MCV [82.0–98.0 fL]	94.5 fL	98.8 fL
PLT [(150–450) × 10^3^/UL]	157 × 10^3^/UL	171 × 10^3^/UL
NEUT ABS # [(2.00–7.80) × 10^3^/UL]	15.06 × 10^3^/UL	2.25 × 10^3^/UL
LYMPH ABS # [(1.00–4.80) × 10^3^/UL]	1.15 × 10^3^/UL	1.14 × 10^3^/UL
SODIUM SERUM [135–145 mmol/L]	132 mmol/L	134 mmol/L
POTASSIUM SERUM [3.5–5.1 mmol/L]	4.0 mmol/L	3.6 mmol/L
CHLORIDE SERUM [98–107 mmol/L]	95 mmol/L	105 mmol/L
HCO3 [22–30 mmol/L]	25 mmol/L	30 mmol/L
ANION GAP [5–19 mmol/L]	12 mmol/L	<4 mmol/L
GLUCOSE SERUM [74–106 mg/dL]	174 mg/dL	97 mg/dL
BUN SERUM [9–20 mg/dL]	23 mg/dL	7 mg/dL
CREATININE [0.66–1.25 mg/dL]	0.8 mg/dL	0.6 mg/dL
CALCIUM SERUM [8.4–10.2 mg/dL]	8.4 mg/dL	7.5 mg/dL
ALBUMIN SERUM [3.5–5.0 g/dL]	2.9 g/dL	2.1 g/dL
PROTEIN SERUM [6.3–8.2 g/dL]	7.4 g/dL	6.2 g/dL
TOTAL BILIRUBIN [0.2–1.3 mg/dL]	1.4 mg/dL	0.7 mg/dL
GOT (AST) [17–59 Intern Unit/L]	29 Intern Unit/L	26 Intern Unit/L
ALK PHOS [38–126 Intern Unit/L]	261 Intern Unit/L	254 Intern Unit/L
GPT (ALT) [0–50 Intern Unit/L]	34 Intern Unit/L	24 Intern Unit/L
C3 [88–201 mg/dL]	101 mg/dL	N/A
C4 [15–45 mg/dL]	12.7 mg/dL	N/A
Cryoglobulins [Negative]	Positive	N/A
ANA [Negative]	Negative	N/A

### 2.2 Case 2

**FIGURE 2:**
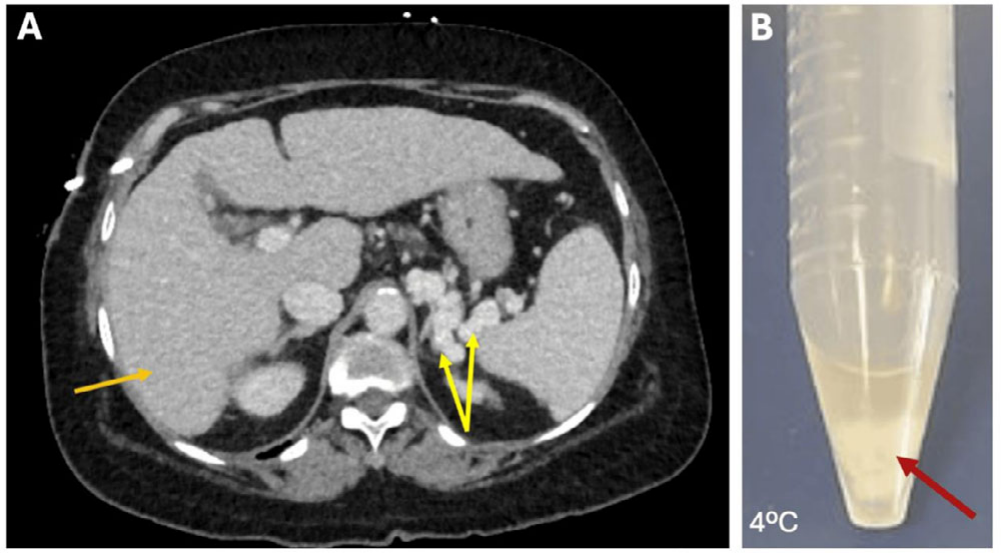
(A) Abdominal computed tomography (CT) showed the presence of the liver with cirrhotic morphology (orange arrow), as well as with multiple left upper quadrant varices consistent with portal hypertension (yellow arrows). (B) Serum sample showing a characteristic cryoglobulin pellet present at 4°C (red arrows).

A 67‐year‐old female with a past medical history of Hepatitis C genotype 1a‐related cirrhosis (Figure 2A) [14] presented with severe fatigue and worsening arthralgias. Of note, she was diagnosed with hepatitis C virus (HCV) on routine bloodwork for elevated liver enzymes one year earlier and had positive history of blood transfusion in Mexico. Her lab results revealed HCV RNA levels to be 676 000 and 5.83. Liver elastography showed CAP value of 254, median liver stiffness of 17.6 kPa with median percentage of 15%, consistent with hepatic steatosis (grade S1) and hepatic fibrosis (stage F4). She was started on HCV treatment with glecaprevir/pibrentasvir, however she discontinued it after 4 weeks due to adverse effects including rash and bilateral lower extremity swelling. She had been self‐medicating for arthralgias with over‐the‐counter steroids. Physical examination revealed palpable purpura. Significant labs are illustrated in Table 3. Further evaluations revealed elevated inflammatory markers, positive autoimmune markers (antinuclear antibody positive, RF positive), with low complement (C4), raising suspicion of mixed CG secondary to HCV history. Her serum cryocrit was found to be positive (Figure 2B). She had the classic Meltzer's triad of severe fatigue, palpable purpura and worsening arthralgias. She was restarted on treatment for HCV infection (with sofosbuvir/velpatasvir) and improvement in the rash and cryoglobulinemic syndrome was observed. No adverse effects were reported. Her sustained virologic response (SVR) after 12 weeks showed negative HCV levels. She was discharged outpatient with follow‐up for rheumatology, gastroenterology and infectious disease.

**TABLE 3:** Laboratory Workup for Case 2.

Serum test [normal range]	Results on initial presentation	Results on discharge
WBC [(4.50–11.00) × 10^3^/UL]	27.63 × 10^3^/UL	6.73 × 10^3^/UL
RBC [(4.20–5.90) × 10^6^/UL]	4.20 × 10^6^/UL	3.35 × 10^6^/UL
HGB [12.0–16.0 g/dL]	13.0 g/dL	10.3 g/dL
HCT [38.0%–47.0%]	39.3%	31.2%
MCV [82.0–98.0 fL]	93.6 fL	93.1 fL
PLT [(150–450) × 10^3^/UL]	210 × 10^3^/UL	171 × 10^3^/UL
NEUT ABS # [(2.00–7.80) × 10^3^/UL]	19.62 × 10^3^/UL	3.41 × 10^3^/UL
LYMPH ABS # [(1.00–4.80) × 10^3^/UL]	5.53 × 10^3^/UL	1.95×10^3^/UL
SODIUM SERUM [135–145 mmol/L]	133 mmol/L	135 mmol/L
POTASSIUM SERUM [3.5–5.1 mmol/L]	5.8 mmol/L	3.9 mmol/L
CHLORIDE SERUM [98–107 mmol/L]	106 mmol/L	106 mmol/L
HCO3 [22–30 mmol/L]	13 mmol/L	22 mmol/L
ANION GAP [5–19 mmol/L]	14 mmol/L	7 mmol/L
GLUCOSE SERUM [74–106 mg/dL]	85 mg/dL	84 mg/dL
BUN SERUM [9–20 mg/dL]	21 mg/dL	3 mg/dL
CREATININE [0.66–1.25 mg/dL]	1.7 mg/dL	0.4 mg/dL
CALCIUM SERUM [8.4–10.2 mg/dL]	10.7 mg/dL	8.4 mg/dL
ALBUMIN SERUM [3.5–5.0 g/dL]	3.3 g/dL	2.4 g/dL
PROTEIN SERUM [6.3–8.2 g/dL]	7.4 g/dL	6.2 g/dL
TOTAL BILIRUBIN [0.2–1.3 mg/dL]	2.6 mg/dL	1.6 g/dL
GOT (AST) [17–59 Intern Unit/L]	36 Intern Unit/L	40 Intern Unit/L
ALK PHOS [38–126 Intern Unit/L]	97 Intern Unit/L	79 Intern Unit/L
GPT (ALT) [0–50 Intern Unit/L]	16 Intern Unit/L	17 Intern Unit/L
C3 [88–201 mg/dL]	95 mg/dL	N/A
C4 [15–45 mg/dL]	11.6 mg/dL	N/A
Cryoglobulins [Negative]	Positive	N/A
ANCA [Negative]	Negative	N/A
Rheumatoid Factor IgG [<6 U]	100 U	N/A
Hepatitis C [Negative]	Positive	N/A

### 2.3 Case 3

A 65‐year‐old female with a past medical history of monoclonal gammopathy of undetermined significance (MGUS, with IgM Kappa monoclonal protein in Gamma region), type II CG vasculitis (monoclonal IgM and polyclonal IgG positive), and membranoproliferative glomerulonephritis (MPGN) confirmed with biopsy presented with severe fatigue. Physical examination revealed lower extremity palpable purpura that had started 3 days earlier. Of note, patient was receiving her first cycle of a rituximab‐biosimilar product a week prior to presentation. Significant labs are illustrated in Table 4. Her infectious work‐up was unremarkable. Her complement (C4) levels were low. Keeping with the suspicion of a cryoglobulinemic flare secondary to use of a biologic agent, pulse‐dose steroids were initiated. No adverse effects were reported. She had clinical and laboratory improvement with resolution of her lower extremity rash and cryoglobulinemic syndrome. She was discharged outpatient with follow‐up for nephrology and hematology‐oncology.

**TABLE 4:** Laboratory Workup for Case 3.

Serum test [Normal range]	Results on initial presentation	Results on discharge
WBC [(4.50–11.00) × 10^3^/UL]	6.51 × 10^3^/UL	12.63 × 10^3^/UL
RBC [(4.20–5.90) × 10^6^/UL]	3.41 × 10^6^/UL	2.79 × 10^6^/UL
HGB [12.0–16.0 g/dL]	10.0 g/dL	8.4 g/dL
HCT [38.0%–47.0%]	31.3%	25.2%
MCV [82.0–98.0 fL]	91.8 fL	90.3 fL
PLT [(150–450) × 10^3^/UL]	167 × 10^3^/UL	235 × 10^3^/UL
NEUT ABS # [(2.00–7.80) × 10^3^/UL]	5.58 × 10^3^/UL	10.86 × 10^3^/UL
LYMPH ABS # [(1.00–4.80) × 10^3^/UL]	0.40 × 10^3^/UL	0.56 × 10^3^/UL
SODIUM SERUM [135–145 mmol/L]	136 mmol/L	129 mmol/L
POTASSIUM SERUM [3.5–5.1 mmol/L]	4.5 mmol/L	4.6 mmol/L
CHLORIDE SERUM [98–107 mmol/L]	103 mmol/L	101 mmol/L
HCO3 [22–30 mmol/L]	26 mmol/L	22 mmol/L
ANION GAP [5–19 mmol/L]	7 mmol/L	6 mmol/L
GLUCOSE SERUM [74–106 mg/dL]	118 mg/dL	129 mg/dL
BUN SERUM [9–20 mg/dL]	43 mg/dL	109 mg/dL
CREATININE [0.66–1.25 mg/dL]	3.8 mg/dL	4.2 mg/dL
CALCIUM SERUM [8.4–10.2 mg/dL]	8.0 mg/dL	7.6 mg/dL
ALBUMIN SERUM [3.5–5.0 g/dL]	2.6 g/dL	2.5 g/dL
PROTEIN SERUM [6.3–8.2 g/dL]	4.9 g/dL	4.6 g/dL
TOTAL BILIRUBIN [0.2–1.3 mg/dL]	1.3 mg/dL	0.3 g/dL
GOT (AST) [17–59 Intern Unit/L]	27 Intern Unit/L	33 Intern Unit/L
ALK PHOS [38–126 Intern Unit/L]	46 Intern Unit/L	53 Intern Unit/L
GPT (ALT) [0–50 Intern Unit/L]	17 Intern Unit/L	35 Intern Unit/L
C3 [88–201 mg/dL]	76 mg/dL	N/A
C4 [15–45 mg/dL]	<8.0 mg/dL	N/A
Cryoglobulins [Negative]	Positive	N/A
ANA [Negative]	Positive (1:160)	N/A
Cryoprecipitate IgG [0‐0]	71 mg/dL	N/A
Cryoprecipitate IgA [0‐0]	26 mg/dL	N/A
Cryoprecipitate IgM [0‐0]	128 mg/dL	N/A

## 3 Discussion

Identifying the true etiology of CG is crucial for selecting effective treatment and minimizing the risk of recurrence, morbidity, and mortality. To discuss the clinical implications and management of CG, it is essential to recognize the wide range of conditions associated with this disorder, as highlighted in our case series and supported by existing literature. It is important to remember that natural history of Type I and mixed CG varies considerably. In type I CG, hyperviscosity and vascular obstruction due to monoclonal immunoglobulin aggregates lead to ischemic complications such as digital ulcers, Raynaud's phenomenon, and thrombosis. Renal and neurological involvement is often linked to underlying hematologic malignancies. The prognosis remains guarded, with a 5‐year overall survival rate of 77%–83% and worse outcomes are seen in patients with the IgG isotype or kidney involvement [15, 16, 17]. In contrast, mixed CG follows a relapsing course with diverse manifestations, ranging from mild (e.g., palpable purpura and arthralgia) to severe organ involvement, including MPGN and neuropathy. In HCV‐related cases, up to 12% of patients may develop B‐cell lymphoma. However, the introduction of direct‐acting antivirals (DAAs) has dramatically improved prognosis, with 5‐year survival rates now reaching up to 75%. Patients with type III CG typically have milder presentations than those with type II disease [18, 19, 20]. Despite these advancements, non‐infectious mixed CG still poses diagnostic and therapeutic challenges, particularly when associated with autoimmune diseases.

Our first case highlights CG in a patient with *S. aureus* septicemia and metastatic colon cancer, illustrating the complex association between infection, malignancy, and CG. Misdiagnosing sepsis‐related CG and relying solely on immunosuppressive therapy can lead to repeated relapses, emphasizing the importance of accurate diagnosis to guide appropriate therapy [21]. *S. aureus*, typically causes acute IE with rapid onset, classic symptoms, and easily identifiable positive blood cultures enabling quick diagnosis. However, when subacute or chronic IE goes undiagnosed for a longer period, it allows time for an immune response to develop, leading to mixed CG as an immunological complication [22, 23]. Reinburg et al. reported a case where IE was identified as the underlying cause of CG, with symptoms improving following antibiotic therapy [21]. In our case, the patient developed mixed cryoglobulinemic vasculitis associated with *S. aureus* bacteremia, and showed symptom improvement with antibiotic treatment, confirming sepsis as the primary etiology.

Among individuals infected with HCV, 40%–60% have circulating cryoglobulins; however, only 5%–15% of these individuals will progress to develop CV [5]. CG, particularly the mixed type (Types II and III), has been frequently linked to HCV, and other infections including hepatitis B virus, and HIV [24, 25]. The second case emphasizes the necessity of vigilance in patients with chronic HCV, who may present with symptoms like fatigue and arthralgias that are often attributed to mixed CG. This is consistent with findings from the literature where HCV is frequently identified as a common cause of mixed CG. Despite antiviral therapy being the mainstay of treatment, cases may arise where patients exhibit intolerance or incomplete response to antiviral medications, requiring additional interventions like rituximab [9]. Our patient had side effects to initial HCV therapy leading to non‐compliance. She then developed cryoglobulinemic syndrome which resolved with initiation of a different HCV treatment regimen,

The onset of CG following rituximab‐based therapy is due to a transient IgM increase, termed IgM flare, which may cause symptomatic hyperviscosity, exacerbate IgM‐related neuropathy, or aggravate CG and other complications. Elevated IgM can persist for months, typically requiring plasmapheresis rather than indicating treatment failure. Prophylactic plasmapheresis is advisable for patients with high baseline IgM (≥4000 mg/dL) before rituximab to reduce flare risks [26, 27]. The third case underscores the importance of monitoring for flares during immunomodulatory therapy, with pulse‐dose steroids proving effective in managing acute flares [17]. Our third patient had IgM Kappa monoclonal protein making her more susceptible to a cryoglobulinemic flare.

The treatment of CG depends on the type, underlying etiology, and disease severity. For type I CG, supportive measures include avoiding cold exposure and using compression garments. Pharmacologic strategies are tailored to the immunoglobulin isotype [28]. In IgM‐mediated disease, rituximab, alkylating agents, and Bruton's tyrosine kinase inhibitors are preferred. IgG‐mediated disease may be managed with bortezomib, anti‐CD38 agents like daratumumab, or immunomodulatory drugs such as lenalidomide. Plasmapheresis is often employed to address hyperviscosity symptoms, especially in IgM‐related cases. Ultimately, treatment should target the underlying hematologic disorder, if present, to prevent disease progression and improve survival [29]. For mixed CG, management focuses on treating the underlying cause and controlling vasculitis. In HCV‐related cases, DAAs such as sofosbuvir/velpatasvir have become the cornerstone of treatment, achieving SVRs and vasculitis remission in most patients. For mild‐to‐moderate disease, these antiviral agents alone may suffice. In noninfectious cases, low‐dose glucocorticoids, colchicine, or methotrexate may be used. Severe manifestations, such as glomerulonephritis, neuropathy, or skin necrosis, necessitate combining DAAs with rituximab and glucocorticoid pulses. Life‐threatening presentations may require additional plasma exchanges. In rare instances of associated hematologic malignancies, regimens incorporating rituximab, fludarabine, and cyclophosphamide may be employed. Patients with refractory disease or those ineligible for DAAs may benefit from rituximab maintenance therapy administered every 6–9 months. Novel therapies, including low‐dose interleukin‐2 and belimumab, show promise for noninfectious mixed CG, especially in refractory cases. Furthermore, targeted treatments such as obinutuzumab (a next‐generation anti‐CD20 agent) are being evaluated in clinical trials [30, 31]. The treatment strategies leading to improvement in all our cases have been summarized in Table 1.

These cases underscore the necessity for a comprehensive and multidisciplinary approach to managing CG. The variability in clinical presentation, as well as the wide spectrum of associated conditions—from infections and malignancies to autoimmune disorders—requires careful diagnostic evaluation and tailored therapeutic strategies. While guidelines are limited, recognizing patterns from case reports and studies can significantly aid in optimizing patient outcomes and advancing our understanding of CG.

In summary, these cases highlight the diversity and complexity of CG and may guide physicians to make opinion‐based decisions with the help of precedents, in the absence of clear guidelines to treat this array of diseases. Although individually tailored treatment strategies will likely be necessary to improve patient outcomes and quality of life in the absence of standard guidelines, we note similarities in treatment patterns between cases described in previous studies. Our study also emphasizes scenarios requiring high index of suspicion for CG in the context of underlying comorbidities. A multidisciplinary approach including hematologists, oncologists, rheumatologists, infectious disease specialists prove essential to enhance our understanding and management of this collection of complex disorders.

## Author Contributions

This paper was conceptualized by Shivangini Duggal, Lakshmi Vaishnavi Prasanna Kattamuri, Michel Toutoungy, Eder Luna Ceron, and Madhumita Rondla. The investigation and review of relevant data and articles were done by Shivangini Duggal, Lakshmi Vaishnavi Prasanna Kattamuri, Michel Toutoungy, Eder Luna Ceron, and Madhumita Rondla. Case analysis was done by Shivangini Duggal, Lakshmi Vaishnavi Prasanna Kattamuri, Michel Toutoungy, Eder Luna Ceron, and Madhumita Rondla. The original draft was written by Shivangini Duggal, Lakshmi Vaishnavi Prasanna Kattamuri, Michel Toutoungy, Eder Luna Ceron, and Madhumita Rondla. Review and editing were done by Angelica Lehker. All authors discussed the findings described in the case and approved the final manuscript.

## Ethics Statement

Our institution does not require ethics committee approval for case series.

## Patient Consent Statement

Patients have been contacted and consented verbally.

## Conflicts of Interest

The authors declare no conflicts of interest.

## Supporting information



Supporting Information

## Data Availability

Data sharing not applicable to this article as no datasets were generated or analyzed during the current study.
