# Supplementary material for: Exploring Cryoglobulinemia's Clinical Odyssey: A Case Series
Source: EJHaem. 2025 Apr 2;6(2):e70029. doi: 10.1002/jha2.70029 (PMC11962755; doi:10.1002/jha2.70029)
Supplement: Supplementary file 1 — Supporting Information [file JHA2-6-e70029-s001.docx]

🔬 **Exploring Cryoglobulinemia’s Clinical Odyssey!** 🩸

Our **case series** showcases three distinct presentations of **cryoglobulinemia**:
✅ **Infection-triggered CG** in metastatic colon cancer
✅ **HCV-related mixed CG** responding to antiviral therapy
✅ **Rituximab-induced CG flare** managed with steroids

Each case underscores the **importance of early recognition, precise diagnosis, and individualized treatment** in managing this complex disorder.

📖 Read more to see how a **multidisciplinary approach** can optimize outcomes! #MedTwitter #Rheumatology #Hematology #InfectiousDiseases
